# Acknowledgement to Reviewers of *Antioxidants* in 2017

**DOI:** 10.3390/antiox7010008

**Published:** 2018-01-10

**Authors:** Antioxidants Editorial Office

**Affiliations:** MDPI AG, St. Alban-Anlage 66, 4052 Basel, Switzerland

Peer review is an essential part in the publication process, ensuring that *Antioxidants* maintains high quality standards for its published papers. In 2017, a total of 103 papers were published in the journal. Thanks to the cooperation of our reviewers, the median time to first decision was 16 days and the median time to publication was 39 days. The editors would like to express their sincere gratitude to the following reviewers for their time and dedication in 2017:
Abdalla, Maher Y.Greenberger, Joel S.Pani, BibhusitaAbourashed, EhabGrimm, MarcusPaoli, PaoloAfolayan, Adeleye J.Grimm, Marcus O. W.Pappas, ChristosAlam, JawedGuido, Luis F.Paramithiotis, SpirosAlmajano, María PilarGünes, CagatayPedrosa, Mercedes M.Amarowicz, RyszardGupta, SanjayPeijnenburg, Willie J. G. M.Anastasaki, EiriniGustin, MikePereira, Olívia R.Anjos, Ofélia Maria Serralha DosHankinson, OliverPetrucci, RitaArroo, RandolphHano, ChristophePhan-Thien, Kim-YenAsahina, KinjiHarasym, JoannaPiemontese, LucaAykin-Burns, NukhetHare, DominicPignitter, MarcBacelar, EuniceHarrison-Bernard, Lisa M.Podszun, MarenBagher, PoonehHarroun, ThadPors, KlausBallmann, ChristopherHarvey, AdamProestos, CharalamposBarba, FranciscoHatano, TsutomuQuinzii, Catarina MBartosz, GrzegorzHernández-Hernández, A.Raha, SandeepBatinic-Haberle, InesHewett, SandraRashidinejad, AliBedse, GauravHitosugi, TaroReis, FlávioBELIN De Chantemèle, Eric J.Hsueh, Yi-HuangRevilla, EugenioBellés, José MaríaHuang, Ting-tingRiccardo, IentileBerry, Ann AndersonHumphries, Kenneth M.Ricelli, AlessandraBeyer, Andreas M.Ichikawa, HiroshiRostagno, Mauricio A.Birringer, MarcIgnatowicz, EwaRouch, AlBohn, TorstenJaiswal, Amit K.Rubis, BlazejBosch, ChristineJarajapu, Yagna PRRupasinghe, Thusitha W. T.Bubb, KristenJensen, Søren KroghRusso, Gian LuigiBurt, DeanJiang, QingRybalka, EmmaCaboni, Maria FiorenzaJin, JinSaisho, YoshifumiCádiz Gurrea, María De La LuzJohnston, CarolSaito, YoshiroCalani, LucaJurewitsch, BrianSalvador, María DesamparadosCanini, AntonellaKao, Shu-HueiSan Martin, CarmenCarmo, Sonia DoKarousou, EvgeniaSansone, ClementinaCarnevale, RobertoKashatus, DavidSantos, Sónia A. O.Carocho, MárcioKauppinen, AnuSatou, RyousukeCartland, SianKavurma, MarySavion, NaphtaliCarvalho, PauloKent, KatherineSayin, VolkanCeleiro, MaríaKhachik, FrederickSchiavone, MarcoChae, HeeyoungKlarskov, KlausSchrag, MatthewChaimbault, PatrickKlein, EricSchröder, KatrinChami, BelalKocot, JoannaSchuck, AlyssaChang, Hsueh-WeiKonstorum, AnnaScognamiglio, MonicaChen, Chun-JungKontogiorgis, ChristosSeca, Ana M. L.Chen, Jih-JungKopjar, MirelaSforza, ChiarellaCheong, Yuen-KiKouretas, DimitriosSgherri, CristinaCole, MarshaKriebardis, Anastasios G.Sharma, AshishCumming, Kristoffer T.Kukula-Koch, WirginiaShidoji, YoshihiroD’Alessandro, AngeloKump, DavidSieber, FritzDall’Acqua, StefanoLaukkanen, MikkoSiegenthaler, JulieDasari, SubramanyamLedo, AnaSilva, RobinDasgupta, SantanuLehtonen, MariSimmonds, Monique S. J.Davinelli, SergioLenaz, GiorgioStagos, DimitriosDe Geest, BartLiby, KarenStamatopoulos, KonstantinosDe Mendonça, Dina I. M. D.Liddell, Jeffrey R.Stauber, Rudolf E.De Paepe, BoelLim, Sang-binStrelec, IvicaDe Vendittis, EmmanueleLin, ShengdaSweazea, KarenDel Giudice, RitaLloret, AnaTait, StephenDeli, Chariklia K.Lloyd, PamelaTanaka, RyouichiDeNicola, GinaLo, CliffordTančinová, DanaDias, Irundika H. K.Luczak, Michal W.Taticchi, AgneseDidonna, AlessandroMa, YanTavares, AnthonyDikalov, SergeyMalemud, Charles J.Taylor, CarolineDi Maio, RobertoMalli, RolandTergaonkar, VinayDonaldson, Robert P.Mancini, InesTieu, KimEcelbarger, Carolyn M.Manor, DannyTiidus, PeterEnache, Teodor AdrianManthou, EiriniTsai, Kun-LingFang, JimMaranchie, JodiTsuji, PetraFatouros, IoannisMarengo, BarbaraTurner, MarkFelder, Robin A.Martínez, SidoniaUlatowski, LynnFeresin, Rafaela G.Martínez-Espinosa, Rosa MaríaVallini, GiovanniFernandes, Ana S.Maruyama, ToruVan Raamsdonk, Jeremy MFernandes, EduardaMcAnulty, LisaVassalle, CristinaFernandez-Twinn, Denise S.Mcmorrow, TaraVeeramani, SureshFine, James BurkeMéndez-Álvarez, EstefaníaVlassopoulos, AntonisFitsanakis, VanessaMendonca, AntonioWang, Horng-DarFocsan, Alexandrina L.Merry, TroyWeber, JohnForini, FrancescaMessina, Concetta MariaWiggs, MichaelForster, BrittaMiguel, GraçaWilliams, KimberlyFoutch, BrianMinato, Ken-ichiroWinocur, EphraimFox-Robichaud, AlisonMonti, Daria MariaWojtovich, AndrewFujii, RitsukoMoreno, SilviaWoodman, TimFulton, David J.Moro, LoredanaWu, Li-ChenGail, WelsbyMorris, WayneWu, Tzu-HuaGangula, PanduMortiboys, HeatherWu, YingGanini, DouglasMuñiz, PilarXin, LingGarcia Vaquero, MarcoMurdoch, ColinXing, XiaohuiGarten, RyanNaylor, KariYang, Shun-ChinGavathiotis, EvripidisNeilson, AndrewYang, YueGentile, CarmineNenadis, NikolaosYee, Callista StephanieGeorgakilas, AlexandrosNickel, AlexanderYoon, Wan HeeGerber, Leonard E.Nieman, David C.Yuan, LiyunGermain, DorisNolan, JohnZarrelli, ArmandoGetty, Kelly J. K.Nolvachai, YadaZavacki, Ann-MarieGhosh, RitaNozik-Grayck, EvaZhang, HuaGieseg, Steven P.O’callaghan, Yvonne C. Zhang, PangzhenGiuffrè, Angelo MariaObana, AkiraZhang, YiqiangGolczak, MarcinOlejar, Kenneth J.Zhou, Huanjiao JennyGolmohamadi, AmirOlson, KennethZłotek, UrszulaGonçalves, Fernando J.Olson, Kenneth R.Zugaza, José LuisGoreham, ReneeOlszewska, Monika A.Zuryn, StevenGoswami, Prabhat C.Ormsbee, Michael
Grant, RossPallardó, Federico V. 


